# Sediment delivery to sustain the Ganges-Brahmaputra delta under climate change and anthropogenic impacts

**DOI:** 10.1038/s41467-023-38057-9

**Published:** 2023-04-27

**Authors:** Jessica L. Raff, Steven L. Goodbred, Jennifer L. Pickering, Ryan S. Sincavage, John C. Ayers, Md. Saddam Hossain, Carol A. Wilson, Chris Paola, Michael S. Steckler, Dhiman R. Mondal, Jean-Louis Grimaud, Celine Jo Grall, Kimberly G. Rogers, Kazi Matin Ahmed, Syed Humayun Akhter, Brandee N. Carlson, Elizabeth L. Chamberlain, Meagan Dejter, Jonathan M. Gilligan, Richard P. Hale, Mahfuzur R. Khan, Md. Golam Muktadir, Md. Munsur Rahman, Lauren A. Williams

**Affiliations:** 1 Department of Earth and Environmental Sciences, Vanderbilt University, Nashville, TN USA; 2 Center for Applied Earth Science and Engineering Research, The University of Memphis, Memphis, TN USA; 3 Department of Geology, Radford University, Radford, VA USA; 4 Department of Geology and Geophysics, Louisiana State University, Baton Rouge, LA USA; 5 Department of Earth and Environmental Sciences, St Anthony Falls Laboratory, University of Minnesota, Minneapolis, MN USA; 6 Lamont-Doherty Earth Observatory, Columbia University, Palisades, NY USA; 7 Haystack Observatory, Massachusetts Institute of Technology, Westford, MA USA; 8 Centre de Géosciences, PSL University/ MINES Paris, Fontainebleau, France; 9 CNRS - Littoral Environnement et Sociétés, La Rochelle University, La Rochelle, France; 10 Institute for Arctic and Alpine Research, University of Colorado, Boulder, CO USA; 11 Department of Geology, University of Dhaka, Dhaka, Bangladesh; 12 Bangladesh Open University, Board Bazar, Gazipur, Bangladesh; 13 Department of Earth and Atmospheric Sciences, University of Houston, Houston, TX USA; 14 Soil Geography and Landscape Group, Wageningen University, Wageningen, Netherlands; 15 Department of Ocean & Earth Sciences, Old Dominion University, Norfolk, VA USA; 16 Department of Environmental Science, Bangladesh University of Professionals, Dhaka, Bangladesh; 17 Institute of Water and Flood Management, Bangladesh University of Engineering and Technology, Dhaka, Bangladesh

**Keywords:** Climate-change impacts, Environmental impact, Climate-change impacts, Geomorphology, Sedimentology

## Abstract

The principal nature-based solution for offsetting relative sea-level rise in the Ganges-Brahmaputra delta is the unabated delivery, dispersal, and deposition of the rivers’ ~1 billion-tonne annual sediment load. Recent hydrological transport modeling suggests that strengthening monsoon precipitation in the 21st century could increase this sediment delivery 34-60%; yet other studies demonstrate that sediment could decline 15-80% if planned dams and river diversions are fully implemented. We validate these modeled ranges by developing a comprehensive field-based sediment budget that quantifies the supply of Ganges-Brahmaputra river sediment under varying Holocene climate conditions. Our data reveal natural responses in sediment supply comparable to previously modeled results and suggest that increased sediment delivery may be capable of offsetting accelerated sea-level rise. This prospect for a naturally sustained Ganges-Brahmaputra delta presents possibilities beyond the dystopian future often posed for this system, but the implementation of currently proposed dams and diversions would preclude such opportunities.

## Abstract (web summary)

The potential for enhanced sediment delivery to the Ganges-Brahmaputra delta exists, but it alone is insufficient to sustain the system. The delta may be resilient to climate change, but only in the absence of dam construction and water diversions.

## Introduction

Only in the past few years have global assessments of river-delta response to accelerated sea-level rise and declining sediment supply utilized datasets more complete^1,2^ than the single mean values often used in earlier studies^3–5^. Most earlier assessments yield grave predictions for the response of deltas to climate change and human-related impacts, and while those concerns are not unfounded, they generally oversimplify complex system behaviors and can thus mask potentially more positive delta scenarios^2^. One risk of such negative but simplified assessments is to foster dystopian narratives that uphold engineering structures or abandonment of vast areas of coastal land as the key responses to climate change, thereby undermining local to regional efforts to maintain sediment supply and preserve land in these low-lying landscapes^3,6^.

A robust supply of clastic sediment is the principal nature-based resource for offsetting relative sea-level rise in river deltas, yet better constraints and data availability on the delivery and dispersal of sediment are needed^7^ to ensure that future delta scenarios accurately account for both dynamic natural-system behavior^8^ and the continuing impacts of development and land-use change. Furthermore, it is increasingly important that coupled human-natural approaches be used to predict delta fate, because as many as 630 million people currently live in regions that may be uninhabitable by 2100 due to relative sea-level rise (RSLR), flooding, and inundation^9^. Recent global delta studies using highly resolved, spatially and temporally varying data inputs indeed suggest that more deltas than generally recognized have been gaining land and that deltas overall may be collectively more robust than previously thought^2,10–12^.

These recognitions are important because the fate of river deltas and their growing populations are closely tied to riverine sediment supply^10^, which has a direct effect on land reclamation efforts, household migration decisions, and the ability to occupy marginal (vulnerable) lands^13–15^. For instance, the relocation of >20,000 Rohingya refugees to a newly emergent tidal island in the Ganges–Brahmaputra river-mouth estuary is a vivid example involving each of these issues^16,17^. In fact, the Bangladesh Delta Plan 2100 proposes numerous coastal barriers (i.e., cross-dams) to trap sediment in the same region and accelerate the growth of new land for potential reclamation and human occupation^18^. The social, engineering, and political challenges of relocating displaced communities to emergent lands notwithstanding^19^, a pre-requisite for coastal land reclamation remains the uninhibited delivery of Ganges–Brahmaputra river sediment and its effective dispersal across the coastal zone.

Among the world’s river deltas, the Ganges–Brahmaputra system presents an important example of deltaic response to climate change and sea-level rise due to its naturally large sediment supply, (currently) limited upstream damming, and an immense basin population of ~500 million people^20^. The basin’s hydrology, climate, and sediment transport are controlled by the seasonal South Asian monsoon, the strength of which varies considerably over decadal to millennial timescales^21–23^. Indeed, both modeling and proxy records document large fluctuations throughout the Holocene, with stronger monsoon circulation and greater precipitation from the early to mid Holocene followed by a general monsoon weakening and reduction of precipitation after ~6 ka (Fig. 2A, B)^24–30^. For the coming century, the most recent IPCC AR5 and AR6 reports continue to predict increased and more variable monsoon precipitation by 2100 under nearly all representative GHG concentration pathways (RCPs)^31,32^. Such increases may be comparable to periods of stronger monsoon earlier in the Holocene, and thus provide a possible analog for future responses. In addition to the strengthening of monsoon precipitation, regional warming may also enhance the melting of Himalayan glaciers and further increase discharge and sediment loads for at least the coming decades or century^33,34^^.^

Observation-based estimates for the supply of sediment currently delivered to the Ganges–Brahmaputra delta range from <500 to >1,100 Mt/yr (Mt = Mega-tonnes or 10^6^ metric tonnes), representing considerable uncertainty and a critical knowledge gap for projecting delta response to sea-level rise^7,35–37^. Looking forward, recent modeling studies by Darby et al.^38^, Dunn et al.^39^ and Higgins et al.^33^ each consider future changes in Ganges–Brahmaputra sediment supply and their potential consequences for the delta. Their findings suggest that historically unprecedented levels of change in sediment supply from the Ganges and Brahmaputra rivers are possible over the next 50–100 years, either through reductions from damming and diversions^33^, increases from strengthened monsoon precipitation and higher river discharge^7,38^, or a combination of these factors^39,40^. The modeled changes, although large (±50–90%), are comparable to other Asian river systems that have experienced major changes in sediment load over the past century^41–43^.

**Fig. 1:**
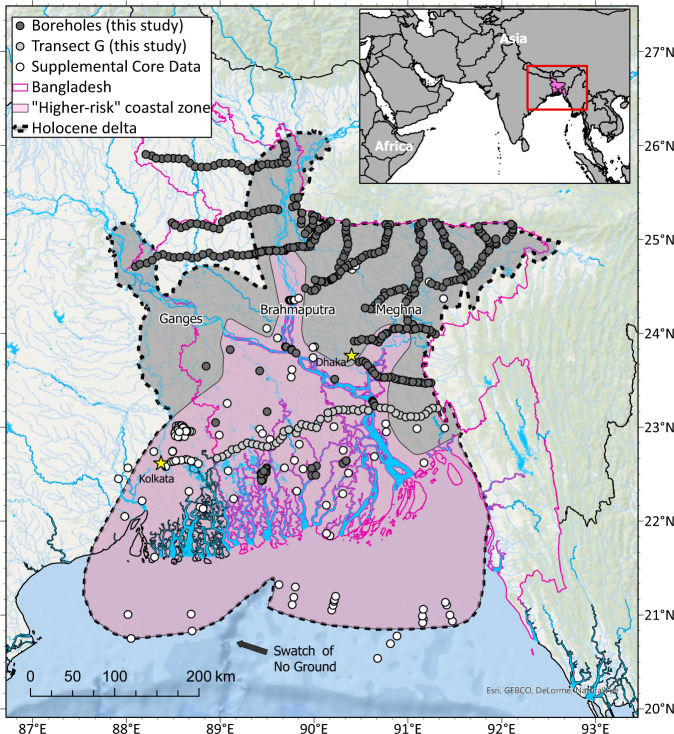
Map of the Bengal basin and the Ganges–Brahmaputra River delta in South Asia showing the study area and and drilling/sampling and data locations. The delta and lower Ganges and Brahmaputra rivers are located in Bangladesh and West Bengal, India. The Holocene delta developed on an incised Pleistocene surface and the boundary of Holocene deposits is shown by the black dashed line. The subaqueous portion of the Holocene delta is located on the shelf and bordered by the Swatch of No Ground canyon and Bay of Bengal to the south. Over 450 boreholes from this study are denoted by the dark and light gray–filled circles^108,131,132^, with the light gray circles demarking Transect G cores shown in Fig. S1. Core and acoustic-flection data from other studies used to define the thickness and extent of Holocene delta deposits are shown by white-filled circles^110–115,117,119,121–123^. The border of Bangladesh is outlined in pink, and the pink-shaded region represents the higher-risk coastal zone based on the model domain used in Akter et al.^91^. World Ocean Base layer is from Esri, GEBCO, NOAA NGDC, HERE, Garmin, and other contributors; the world countries shapefile is from Esri, Garmin International, Inc., and the U.S. Central Intelligence Agency (The World Factbook); and the hydrology shapefiles (World Water Bodies and World Linear Water) are from Esri and Garmin International, Inc.

Toward a validation of such modeled projections of future sediment delivery to the Ganges–Brahmaputra, we present a highly resolved, mass-balanced sediment budget that: (a) utilizes over 6000 new field-based measurements from 500 locations (Fig. 1) to provide (b) ground-truthing for modeling studies and (c) a holistic view of river system connectivity from the Himalayan source terrains to the Bengal deltaic continental margin^44,45^. The budget is derived from previously unpublished borehole data on the volume, mass, grain size, and provenance of Holocene sediment stored in the Bengal basin and are supplemented by a compilation of additional data from over 15 other studies (Tables 1, 2, and S1). In addition to greatly improved spatiotemporal resolution, this new work is distinct from previous Ganges–Brahmaputra mass budgets^46,47^ that lacked any information on the river source (i.e., provenance) or grain-size distribution of stored sediments. Both of these new data types yield valuable results on total bedload contribution to the delta and the varying and asymmetric delivery of sediment from the two main rivers in response to climate change. To our knowledge, no mass balance of comparable detail and longevity exists for a major river delta, thus providing an unparalleled perspective on natural-system response to climate change and an opportunity to ground-truth modeled responses under future climate scenarios.

**Table 1:** Average mass storage rates and grain-size distributions for deposits stored on the Ganges–Brahmaputra delta over the last 12 kyr

Years	Silt and clay (Mt/yr) (<62.5 μm)	Very fine-fine sand (Mt/yr) (62.5–250 μm)	Medium-coarse sand (Mt/yr) (250–1000 μm)
6–0 ka^a^	389	294	162
8–6 ka^a^	425	519	309
10-8 ka	505	824	671
12–10 ka	171	277	371

Highest rates of sediment delivery and storage in the delta occur between 10 and 8 ka and correspond with a sandier sediment load.

^a^Offshore mass distributed with 75% of offshore material contained in the 6–0 ka unit and 25% of offshore material contained in the 8–6 ka unit.

**Table 2:** Average mass storage rates and percent contribution for each river system through the Holocene

Source	Storage rate (Mt/yr)	Percent contribution
**6–0 ka**		
Ganges	426	50.4%
Brahmaputra	377	44.7%
Mixed G + B	7	0.8%
Other	35	4.1%
Total	845	
**8–6 ka**		
Ganges	308	24.6%
Brahmaputra	726	58.0%
Mixed G + B	17	1.3%
Other	202	16.1%
Total	1253	
**10–8 ka**		
Ganges	413	20.7%
Brahmaputra	1119	55.9%
Mixed G + B	128	6.4%
Other	340	17.0%
Total	2001	
**12–10 ka**		
Ganges	116	14.2%
Brahmaputra	512	62.6%
Mixed G + B	16	2.0%
Other	174	21.2%
Total	818	

**Fig. 2:**
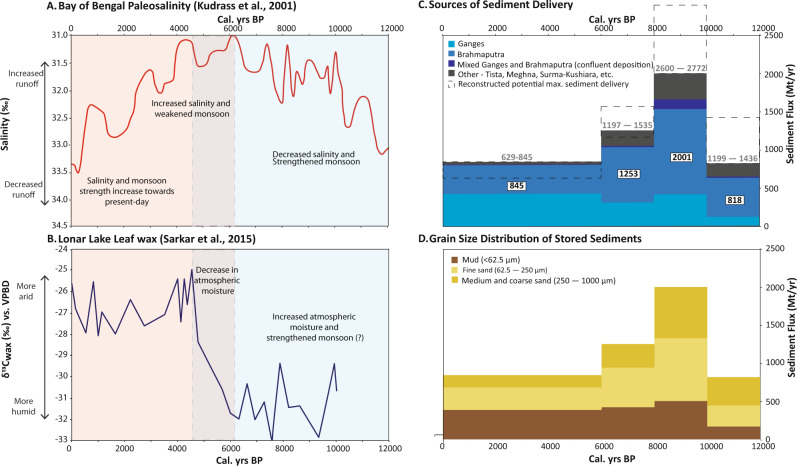
Holocene variability in sediment mass and caliber corresponds with changes in paleosalinity and atmospheric moisture as proxies for freshwater discharge and monsoon precipitation, respectively. **A** A paleosalinity record from sediment cores collected in the northern Bay of Bengal^26^ provides a proxy record for an enhanced early Holocene monsoon, when runoff and river discharge increased and lowered salinity of the surface mixed layer. Salinities decrease after 6 ka under weakening monsoon precipitation and reduced (but still large) river discharge. **B** Terrestrial records of leaf-wax stable carbon isotopes from lacustrine sediments in Lonar Lake, central India^25^ provide evidence for increased atmospheric moisture during the Holocene Climatic Optimum (~9–5 ka), with sharply decreasing moisture levels with monsoon weakening after ~6 ka. **C**, **D** Sediment mass and caliber varies over the Holocene. In the early Holocene, most sediment was deposited by the Brahmaputra River, followed by the Ganges. For the last 6 kyr, the two rivers have deposited a roughly equivalent mass of sediment on the delta but with few deposits reflecting mixing between them. Other tributary sources have locally contributed sediment to the delta, but the amount is comparatively minor. Observed sediment delivery rates over the Holocene are denoted in white boxes and the reconstruction of the maximum potential sediment load based on bypassing estimates are shown in gray text within the dashed boxes.

The Brahmaputra is the main contributor of sediment in the early-mid Holocene (12–6 ka), while the Ganges delivers most of the sediment over the last 6 kyr. Storage rate data are plotted in Fig. 2C.

Here we explore the validity of future sediment-delivery projections by comparing them with previous responses of the Ganges–Brahmaputra sediment load to Holocene climate change and monsoon strength. To do so we use the new sediment database to carefully quantify the mass and grain-size distribution (caliber) of sediment sequestered to the delta and how that mass was distributed and stored to build the delta through major changes in climate and sea level. Finally, we compare the reconstructions of sediment delivery and storage with the sediment demands that will be required for the delta to offset projected increases in sea-level rise for the coming century.

## Results and discussion

### Total delta sediment composition

Reconstructed sediment storage rates from the stratigraphic record represent minimum total riverine sediment loads (bedload and suspended load) delivered to the Ganges–Brahmaputra delta. Unless otherwise noted, sediment delivery will be used to refer to sediment loads reconstructed from stored Holocene sediments. The total mass of sediment stored in the Ganges–Brahmaputra basin over the Holocene (12–0 ka) is >1.2 × 10^7^ Mt (Table 1), which averages to a minimum mean delivery rate of 1101 Mt/yr and is comparable to the oft-cited modern load of ~1100 Mt/yr. The grain size of sediment stored in the delta is approximately equally apportioned across muds (<62.5 μm – 34%), very fine to fine sand (62.5–250 μm – 38%), and medium to coarse sand (250–1000 μm – 28%) (Table 1). For this study, we conservatively classify sediments <250 μm (muds to fine sand) as suspended load and larger particles 250–1000 μm as bedload^48,49^. These delineations result in Holocene delta deposits comprising 72% suspended load and 28% bedload sediments, considerably exceeding not only the 10% bedload transport rate often presented in the literature where even high estimates are typically placed at 20%^49–52^. The significance is that much of the long-term construction of the delta has been through the aggradation of bedload sand, which is highly susceptible to upstream trapping in reservoirs and thus an important consideration for future management of the delta^41^. Moreover, the bedload plays a disproportionate role in the growth of bars and other locally elevated river topography upon which many village communities are sited.

### Variable Holocene sediment storage

Despite similarity between the average modern and Holocene rates for sediment reaching the delta, sediment delivery over millennial timescales varies by >200% from lowest delivery rates of 845 Mt/yr through the mid-late Holocene (6–0 ka) up to maximum delivery rates in the early Holocene of 1253 Mt/yr (8–6 ka) to 2000 Mt/yr (10–8 ka) (Table 1; Fig. 2). Fluvial sediment began aggrading in the Bengal basin beginning ~12 ka at a rate of ~820 Mt/yr (12–10 ka), but delivery increased to at least 2000 Mt/yr (double the modern load) during the period of strongest monsoon from 10–8 ka. These results confirm previous findings^47^ that the Ganges and Brahmaputra rivers carried a sediment load at least twice as large as the modern for over two millennia. This large supply supported aggradation on the delta sufficient to offset very high rates of sea-level rise that averaged >1 cm/yr over this period. These high early Holocene rates of sediment delivery and sea-level rise are comparable to those anticipated for the next century based on the Darby et al.^38^ modeling for sediment supply and IPCC projections for sea-level rise^53^. These similarities between our paleo-reconstructions and the modeled futures of other studies highlight a potential scenario of sediment supply whereby the delta may be able to maintain itself against increases in sea-level rise over the next century.

Emphasizing that our budget reconstructions represent minimum supply rates, the portions of sediment load that bypassed the delta and transported to the Swatch of No Ground canyon and deep-sea Bengal Fan^54,55^ are not included in the Holocene sediment delivery rates. Such sediment bypass would have been greatest from 12–8 ka when the river channels were constrained to their lowstand valleys and discharged directly to the canyon head. Simple reconstructions of mud deposition in the Swatch of No Ground and active channel of the Bengal Fan^56–58^ suggest that as much as another ~200 Mt/yr of sediment may have been delivered by the rivers in the early Holocene. After 8 ka, though, most sediment delivered to the Bengal margin was efficiently trapped in the Ganges–Brahmaputra delta and thus accounted for in our sediment budget; this sequestration of sediment in the delta is well reflected by the abrupt drop in sedimentation on the Bengal Fan after 9 ka, despite continued high river discharge^56–58^.

In addition to variation in total mass delivered, the sediment loads between 12–10 ka and 10–8 ka are considerably coarser than the Holocene average, comprising ~37% medium to coarse sand that was presumably transported as bedload (Table 1). In contrast, the proportion of med-coarse sand stored in the delta decreases to ~21% after 8 ka. Compared with modern estimates, the early Holocene values are considerably higher than the 10–20% bedload fraction often assumed for the total Ganges–Brahmaputra sediment load^49–52^, although one modern study does cite a bedload fraction of ~35% for each river that is similar to our early Holocene fractions. Regardless of the exact value, our results emphasize that the importance of sand delivery by these large, braided rivers for future delta stability should not be undervalued, particularly in context of the massive sand mining that is occurring in the Ganges–Brahmaputra and other rivers worldwide^59,60^ and the effective trapping of sand in upstream reservoirs^61^.

### Ganges and Brahmaputra sediment provenance

Using bulk Sr concentration of stored sediments, which has been shown to be a reliable indicator of river source in this system^62–64^ (Fig. S1), we have quantified the fraction of sediments delivered to the delta by the Ganges, Brahmaputra, and smaller local rivers (Table 2). Results for the Holocene show that 52% of sediments were sourced by the Brahmaputra, 35% by the Ganges, and the remaining 13% by local rivers including the Tista River in northwest Bangladesh and others draining the Indo-Burman fold belt and Shillong Massif to the east (see Methods for further details). Both major rivers demonstrate considerable variability in sediment delivery throughout the Holocene, but responses are most pronounced for the Brahmaputra catchment (Table 2 and Fig. 2). Since 10 ka, the long-term averages for Ganges sediment load ranges from 308 to 426 Mt/yr, which is comparable to the variance in modern load estimates of 300–450 Mt/yr^37,65,66^. This suggests that Ganges discharge has been relatively stable even with considerable regional climate variability, a finding that is consistent with the similarly modest response projected by Darby et al.^38^ under future climate scenarios. Note that the low Ganges value of 116 Mt/yr stored from 12–10 ka (Table 2) is likely an underestimate due to sediment bypassing, as sedimentation on the Bengal fan remained high at this time^56,58^.

In contrast, sediment load for the Brahmaputra has been highly variable over the Holocene, with a fourfold range from a high of 1119 Mt/yr at 10–8 ka to a low of 377 Mt/yr from 6–0 ka. Relative to the Brahmaputra’s modern load of 500–650 Mt/yr, these values represent a long-term doubling and halving over the Holocene, respectively, reflecting acute response to monsoon strength and related sediment production and transport processes. As for the Ganges, the magnitude of response for our Brahmaputra reconstructions is similar to the projections made by Darby et al.^38^, lending confidence to their results of increased, but differential, future sediment transport for each river. In particular, the differing response of Ganges and Brahmaputra sediment load to climate change will remain highly relevant to managing the delta, given that the largest sediment-source areas for the two rivers lie in India and China, respectively.

### Response to monsoon variability

The modeling of river discharge under future climate change suggests that increases in Ganges–Brahmaputra sediment load of 34–60%^38^ are possible, which would be a substantial increase in sediment delivered to the delta and thus a potentially important buffer against accelerated rates of sea-level rise. These modeled values are supported by our findings from the early Holocene period (12–8 ka) of strengthened monsoon, when fluvial sediment loads were higher for both river systems compared to periods of weaker monsoon from 8–0 ka. Over this full range of monsoon conditions, the Brahmaputra sediment load varied from 377–1119 Mt/yr (i.e., a ±2-fold difference from modern), whereas the Ganges varied only 308–426 Mt/yr (i.e., a range comparable to modern estimates). Note we do not include here the low Ganges value of 116 Mt/yr for the 12–10 ka period due to presumed sediment bypassing at this low sea-level stage.

Our sediment budget reconstructions are also consistent with the results of Darby et al.^38^ further suggesting that the Brahmaputra River catchment is considerably more sensitive to changes in climate, and that during wetter periods it is the more dominant source of sediment to the delta. Importantly, there is no overlap in methods between our paleo-reconstructions and the forward-looking projections of Darby et al.^38^, suggesting that the comparable results provide a reasonable reflection of Ganges–Brahmaputra system dynamics. Among the reasons for such an acute response from the Brahmaputra relative to the Ganges is the catchment’s proportionally smaller lowland area^38^ that limits its capacity for sediment storage. This reduced buffering capacity means that variations in sediment yield from the Brahmaputra catchment reach the delta more quickly and with less attenuation than those from the Ganges catchment^44^. Another factor contributing to acute response of the Brahmaputra sediment supply to climate is the catchment’s large area of relatively arid and sparsely vegetated highlands in Tibet, where modest increases (decreases) in the water budget can drive much higher (lower) erosion rates^67^. Moreover, recent (2003–2008) loss of glacial elevation in the Himalayan catchments^68^ introduces glacial melt water as another source of increased discharge and sediment delivery, although persistence of this contribution may wane beyond 2100 as ice extent contracts^3^.

In contrast to the Brahmaputra, the Ganges catchment lies at the center of the South Asian monsoon system with robust precipitation and vegetation across the Himalayan front range, which is where most sediment and water discharge are generated. The Ganges also hosts a vast foreland filled by alluvial mega-fans that are capable of storing sediment; however, their ability to buffer short-term changes in sediment supply is limited because many fan channels in the Ganges basin are incised and decoupled from the adjacent plains, or are otherwise heavily embanked like the Kosi River fan^69^, causing greater downstream bypass of sediment^52,70^. Indeed, during the early Holocene when the monsoon was strong, the Ganges system incised and remobilized megafan sediments that had aggraded in the early post-glacial period^70–72^. These phenomena in the Ganges catchment support results from models indicating that variable water supply (e.g., due to fluctuations in monsoon strength) can cause periods of aggradation when water flux decreases and periods of incision when water flux increases^73^. Overall, the Ganges fan systems serve to buffer variance in sediment supply as compared with the Brahmaputra that has proportionally less lowland storage area, which is well reflected in both our Holocene reconstructions and the Darby et al.^38^ scenarios for the next century.

Given interest in the potential increase in sediment load under future climate scenarios, it is important to also consider the timescales at which sediment stored in the upper catchment may be remobilized from hillslope, valley, and floodplain settings and delivered to the delta. Our paleo-reconstructions do not provide great precision on those timescales, but evidence from both the Ganges–Brahmaputra and the Indus delta systems show that changes in the supply of riverine sediment load respond to monsoon precipitation at timescales less than the sub-millennial resolution of radiocarbon-dated stratigraphic sections^47,74^. Such short response times would indeed be consistent with hydrologically driven changes in transport and an abundance of available sediment (i.e., a transport limited system)^73^. Indeed ~95% of the Ganges–Brahmaputra sediment load is delivered during the summer monsoon and resulting period of high river discharge (May–October). In this context, more precipitation over the catchment readily remobilizes abundant sediment stored as hillslope regolith and alluvial deposits in the upper catchment. Modern examples also appear to confirm rapid sediment transfer from upland source areas to the Bengal margin, perhaps best exemplified by the decadal-scale transport and delivery of Brahmaputra bedload introduced by 100s of landslides generated in the major 1950 Assam earthquake (M 8.6)^75,76^. Similarly, HydroTrend, the climate-driven hydrological water balance and transport model used by Darby et al.^38^ to consider future basin response to increased precipitation, does not suggest any significant lag time for the delivery of Ganges–Brahmaputra sediment to the delta^77^.

### Increasing sediment supply to support increasing sediment need

**Fig. 3:**
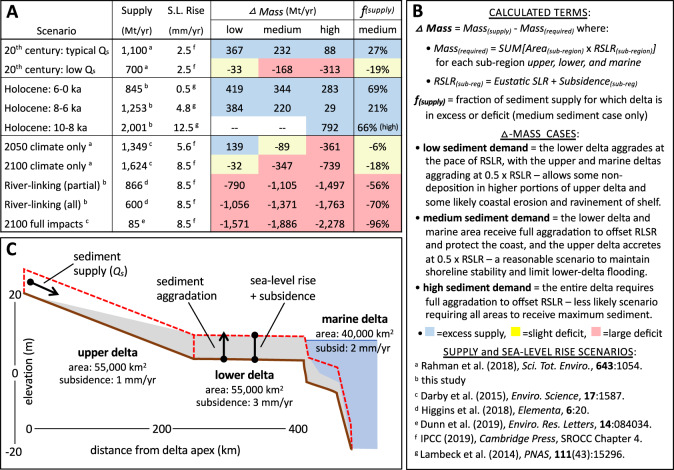
Modern, Holocene, and future mass-balance calculations comparing rates of sediment delivery against the mass required to offset sea-level rise and subsidence. **A** Summary of the mass-balance scenarios, showing input values for sediment supply and sea-level rise and the calculated outputs for *Δ Mass* and *f*_(supply)_. Calculations of required mass for each scenario can be found in Table S4. **B** An explanation of the calculated terms, the conditions for each *Δ Mass* case, and the data sources are provided along with **C**, a schematic diagram of the mass-balance model for the delta. The Holocene: 10–8 ka scenario considers only the high sediment demand case, as the whole delta aggraded in response to rapid sea-level rise during this period.

**Fig. 4:**
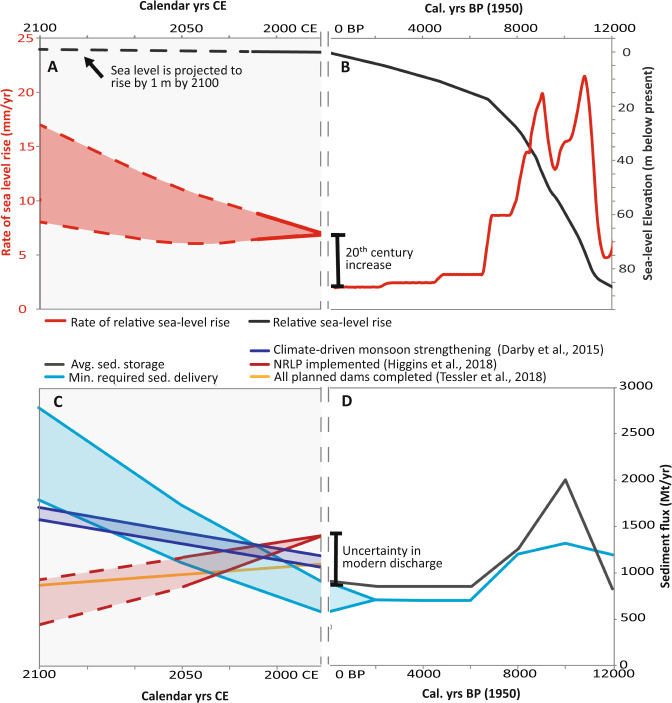
Holocene and predicted future changes in sea level and sediment delivery to the Ganges–Brahmaputra. **A** Comparison of future changes in sea level (shown relative to 1992 CE)^133^ to **B** Holocene changes in global sea level (shown relative to EGM96)^126^. Local sea level (not shown)^134^ and relative sea level is expected to rise by ~1 m by 2100 (blue line, **A**). **C** In the future, predicted changes in monsoon strength^135^ and river damming with the National River Linking Project (NRLP)^33,40^ indicate that sediment fluxes reaching the delta may increase or be reduced. Minimum required sediment delivery is calculated using the whole-delta area (Table S4), so less sediment delivery may still sustain the at-risk portions of the delta against rising sea level. **D** The rate of sediment storage on the delta exceeded the necessary sediment required to keep pace with sea level between 10–8 ka, and for the last 8 ka, whole-delta storage rates have closely matched or exceeded sediment requirements contributing to progradation of the delta. Ultimately, changes in sediment delivery to the delta are likely to occur from river damming^33,40^, sand mining^59,60^, strengthening monsoon^136^, and anthropogenic climate change^135,136^.

To better understand the implications of varying sediment load and rates of sea-level rise on delta sustainability, we have produced mass-balance estimates under a variety of past, present, and future scenarios (Figs. 3 and 4). The *Δ Mass* rates (Mt/yr) represent the annual excess (>0) or deficit (<0) supply of sediment for each scenario, and the *f*_*(supply)*_ is the fractional excess or deficit of delivered sediment specifically for the medium-demand scenario. Results show an excess of sediment delivery under most natural conditions during the Holocene and modern, even during periods of rapid sea-level rise comparable to the rates anticipated for the coming century (5 to 12.5 mm/yr) (Figs. 3 and 4). The modern mass delivery also readily meets demand using the most often reported sediment load value of 1100 Mt/yr, but the delta would be facing a measurable deficit if the occasionally cited value of 700 Mt/yr were correct^35–37,65^. Given the Ganges–Brahmaputra delta’s persistent growth in land area over the last few decades and centuries^78–80^, the higher value of 1100 Mt/yr appears to be more accurate. For the future, unabated sediment delivery in the 2050 and 2100 climate-only scenarios yield a *f*_(supply)_ ranging from a slight to moderate deficits of 7–20%, meaning that erosion rates and land loss may be correspondingly slow and provide valuable time for mitigation strategies to be implemented. These mass-balance results (Fig. 3) are consistent with persistent growth of the delta from Holocene to modern and are not simply optimistic calculations. Rather, these reconstructions and future scenarios (Figs. 3 and 4) emphasize natural resilience of the Ganges–Brahmaputra delta linked to the region’s robust monsoon precipitation and high sediment yield from the tectonically active Himalayan Mountains.

Evidence of a robust and resilient Ganges–Brahmaputra delta has received less attention than scenarios considering a heavily engineered future, where major sediment reductions and high rates of land loss are likely—yet both outcomes are plausible, and the eventual pathway is more contingent on the decisions of policy makers than on climate change. Indeed, our mass-balance estimates (Fig. 3) show massive sediment deficits of more than 100% to over 2000% under all future scenarios that consider widespread dam construction and water diversions^38–40^. These futures are the ones that will more aptly compare the Ganges–Brahmaputra delta with the catastrophic land loss already occurring in the Mississippi, Nile, Indus, Mekong, and other deltas fed by heavily dammed rivers^5,81,82^. Thus, the Ganges–Brahmaputra delta is not doomed to drown under climate change—it is doomed to drown under scenarios of anthropogenic reductions in the sediment delivery needed to sustain the delta^83^.

Many damming and diversion plans for the Ganges and Brahmaputra rivers have been developed but not yet widely implemented^33^, thus presenting a window of opportunity to better steward the delta future. A major risk of not acknowledging the Ganges–Brahmaputra system’s intrinsically robust sediment supply and potential resilience against rising sea level is that environmental managers will otherwise plan for the dystopian future of a drowning landscape and the development of policies that steer toward this anticipated outcome of ruin^6,84^.

Although the potential for considerably enhanced sediment delivery to the Ganges–Brahmaputra delta exists, it alone would still be insufficient to sustain the system. Rather, delivered sediment must also be allowed to freely disperse to regions of the delta where it is needed to offset subsidence and rising sea level. Within the delta itself, widespread embankments in the tidally influenced coastal zone already limit such sediment delivery to poldered islands^83,85^ and have exacerbated local sea-level rise^86,87^. Potentially sustainable efforts to manage sediment accretion in these embanked polders are underway but still face implementation challenges^10,88,89^.

In contrast, in natural areas of the delta such as the Sundarbans mangrove forest, a protected UNESCO World Heritage site, and the Ganges–Brahmaputra’s Meghna rivermouth estuary, unhindered sedimentation is rapid and is readily keeping pace even with accelerated effective sea-level rise (0.5–1.5 cm/yr)^83,90,91^. In fact, in the rivermouth estuary, low barriers (i.e., cross dams) built across shallow waterways have been effective at trapping sediment and rapidly converting open water to emergent intertidal and supratidal lands. Such land reclamation projects are a key part of Bangladesh’s plans for mitigating the effects of sea-level rise^18^, but the success will require the continued uninhibited delivery and dispersal of sediment. Even under scenarios of enhanced sediment delivery, maintaining these natural morphodynamic processes will remain an essential ingredient for long-term sustainability of the delta, which cannot be fully managed or hardened given its size, complexity, and large mass and energy inputs^8,92^.

### Anthropogenic threats to future sediment delivery

Despite projected increases under increased monsoon precipitation, anthropogenic factors may yet dominate the system-scale responses and drive sediment loads to be considerably lower. Specifically, plans across South Asia for hydropower development and interbasinal watershed management portend major reservoir construction and water diversions in the coming century^33,93^. Modeling of the impact of these activities on the river basins suggests moderate (30%) to extreme (88%) decreases in sediment discharge under a range of plausible river-management scenarios^33,39^ (Fig. 3). Existing studies focus on the impact of India’s National River Linking Project (NRLP) and hydropower projects in Nepal, both of which disproportionately affect the Ganges river system^33,39^. No study has yet assessed the potential impact of China’s proposed Motuo hydropower project that would be a run-of-the-river dam of the Yarlung Tsangpo^93,94^, which is a primary water and sediment source estimated to supply 50% of the Brahmaputra’s downstream sediment load.

Such uncertainty in the Ganges–Brahmaputra sediment budget hinders the ability to create sustainable development plans^7^ and makes it possible for upstream decision-makers to justify continued dam construction and water diversions^95–98^ at the expense of downstream sediment supply to the delta^95,99^. Reduced sediment supply will threaten delta stability, particularly if loads decrease below those needed to maintain the delta surface above sea level^88,90^ (Fig. 3). In other mega-deltas like the Mekong and Mississippi, reduced sediment loads from damming and sand extraction have already led to increased saltwater intrusion, decreased soil fertility, loss of land area with rising sea level, and elevation deficits that increase susceptibility to flooding and storm surges^5,81,82^.

Amidst typically negative risk assessments of the Ganges–Brahmaputra delta, it is rarely acknowledged that the system has persistently gained several km^2^/yr of land-surface area for at least the last several centuries, a pattern that continues today without any sign of waning^83,92^. With the next century of climate change, models indicate that the delivery of sediment to the delta will not only persist but likely increase considerably, with the potential to offset accelerating rates of sea-level rise^7,38^. Such projections are consistent with the Holocene sediment reconstructions presented here (Figs. 2 and 3), and these results collectively suggest that the Ganges–Brahmaputra delta may be among the world’s most naturally resilient large coastal systems.

The magnitudes of sediment delivery indicated by our field-based reconstructions and the modeled natural scenarios are very similar, suggesting that the results are reasonably robust; however, even if future increases in sediment load are less than suggested here, a key point is that any increase in sediment supply has the potential to mitigate the impacts of sea-level rise and delay delta drowning. Realizing this potential, though, not only depends on downstream policy decisions that affect the distribution and fate of this sediment in the delta, but also the effects of upstream sediment diversions or retention that threaten the ability of even the wisest downstream policies to mitigate coastal land loss. With global warming and sea-level rise baked into the climate system for centuries, any factors that can delay or reduce impacts, such as increased sediment delivery, may be critically important for effective and manageable human responses.

### Importance of perception to delta futures

Increased sediment delivery under a strengthened monsoon supports the possibility for a more favorable Ganges–Brahmaputra delta outcome under a majority of future climate scenarios. These natural-system responses to climate variability support conclusions from the modeling of future scenarios^38,100^ and modern field-based studies^83,90^ that the delta is not inherently doomed to drown. Instead, proper management of sediment resources, particularly within at-risk coastal regions^85,88^, may provide security for coastal populations and livelihoods often presented as unviable under future climate scenarios^6,13,84,101^. However, the persistence of a doomed-to-drown narrative for Bangladesh may perpetuate dystopian views in which particular regions of the delta or livelihoods are considered unsustainable and thus become subject to maladaptive policies^84^ and may remove attention from the importance of international agreements to coordinate the transport of sediment across national boundaries. Such perceived threats shift the focus of mitigation projects toward the strengthening or expanding of hard infrastructure, approaches that have already disrupted sedimentation processes and undermine the delta’s natural resilience to sea-level rise^8^. The resulting impacts may exacerbate the displacement of households and increase migration away from these supposedly unsustainable coastal locations^6^. Although hard-engineering responses may continue to be part of a sensible coastal management plan, adopting them under threat of a bleak future may steer policies away from more nature-based solutions and other sustainable development policies that would bolster long-term stability of the coastal zone through the effective management of sediment delivery and dispersal.

Upstream of the delta, though, a key concern is how the supply of sediment reaching the delta will be impacted by anthropogenic activities in the catchment basin. In other words, any natural increase in sediment delivery resulting from increased monsoon precipitation^38,100^ will compete with anthropogenic decreases caused by the construction of proposed dams and water diversions upstream of the delta^33,39,88^ (Figs. 3 and 4). Managing these different factors in order to maintain a sustainable delta requires continued diplomatic and scientific focus on the transboundary transport of sediment—focus that is difficult to maintain if a dystopian narrative dominates public discourse. In all, the work presented here and by others^2,83^ offers the possibility for a more optimistic future that is often absent in the literature and media coverage of the Ganges–Brahmaputra delta. The emerging narrative on fate of the delta shifts somewhat away from climate change and more toward the sustainable management of water and sediment resources, both in the upstream catchment and across the delta itself. While there are still significant issues with regard to the uneven distribution of sediment across the delta^102^, the total available sediment could be sufficient for continued sustainability of the delta system as a whole. The caveat is that the delta may only be more resilient to climate change in the absence of major dam construction and water diversions upstream of the delta and major disruptions to the effective delivery and dispersal of sediment within the delta.

## Methods

### Data source

The sediment budget is produced from a largely unpublished dataset of sediment grain-size and geochemistry measured on 6100 sediment samples from 455 boreholes collected by the authors and colleagues between 2011–2021. The boreholes have a maximum depth of 95 m and were collected in 23 transects across the delta. The stratigraphy for about half of these cores (~200) has been published in five previous papers^103–107^, the radiocarbon ages are published in Grall et al.^108^, and local grain-size and mass-balance for one sub-basin of the delta published in Sincavage et al.^109^. These data are supplemented by widespread core and seismic data previously published by refs. ^110–124^. These supplemental data extend across the entire Bengal Basin, including the continental shelf and West Bengal, India where we do not have samples. The complete compilation of data from these sources adds 3720 sites to the more detailed results from our 455 cores.

### Field and laboratory methods

Sediment samples for the cores in this study were collected at 1.5-m depth intervals and photographed, described, and packaged in the field^104–106^. In the lab, every third sample with depth as well as samples at sedimentologic contacts were analyzed for grain-size distribution using a Malvern Mastersizer 2000E particle size analyzer at ¼ ϕ intervals from clays (0.49 μm) to coarse sands (1000 μm). Samples were also analyzed for bulk geochemistry by X-ray fluorescence using a portable Thermoscientific Niton XL3 Analyzer (pXRF), which returns information on composition of both major and trace elements in the sediments. Strontium concentrations from the XRF results are used to document sediment provenance following published methods^62^, effectively distinguishing sediment deposited by the Ganges, Brahmaputra, mixed Ganges–Brahmaputra, or other local river sources^62–64^ (Fig. S1). Sediments having higher bulk Sr values >130 ppm are derived from the Brahmaputra basin, which drains mafic batholiths along the Tsangpo suture zone, compared with lower bulk Sr values <110 ppm for the Ganges (in central and western areas of the delta) or local sediment sources in the north (Tista River), northeast (Shillong Massif), and east (Indo-Burman Fold Belt). Of the 6100 samples analyzed to date for grain size and geochemistry, over 4000 are Holocene-aged and included in this study.

### Mapping

Mapping of Holocene Ganges–Brahmaputra delta deposits was performed using ArcGIS. The Holocene-Pleistocene boundary depth was identified using sediment grain size, color as a proxy for oxidation state, and radiocarbon data from the aggregated dataset^103–106,108,109,116^ and supplemented with depth data from cores, wells, and seismic data in reports and previously published studies^110–123^. Simple co-kriging was used to create an interpolated prediction surface of the depth to the Pleistocene boundary from both the hand drawn contours and depth information from >950 core sites and hundreds of kilometers of seismic lines from many studies^110–123^. To synthesize the data from these 6000+ samples and to control for variance related to regional tectonics and structure, antecedent Pleistocene topography, and the backwater and coastal transition, we partitioned the delta into 9 physiographic and depositional regions based on similarities in processes and inputs (see Fig. S2). Several of these regions are parsed further into sub-regions, largely based on the core-data distribution, to increase the sediment budget resolution and to account for downstream fining and fluvial depositional processes. The main provinces include the Jamuna valley, Ganges valley, Sylhet basin, Meghna valley, Fold Belt, Madhupur Terrace, West Bengal, Interfluve, and Offshore. We then regroup these sub-regions into valleys (Jamuna valley, Ganges valley, Meghna valley), interfluves (Madhupur Terrace, Paleo-interfluve, Fold Belt, West Bengal), and basins (Offshore, Sylhet Basin). To better understand the subsurface stratigraphy, we calculated the grain-size distribution of all samples by 5-m depth bins within spatiotemporal units and then multiplied by the total sediment mass in that unit to yield the sediment-mass grain-size distribution by depth for each region (*n* = 251; see supplemental materials).

Due to spatially varying subsidence rates and temporally changing eustatic sea-level rise that control accommodation in the delta, simple depth conversions do not correlate well with age of the deposits. In other words, establishing well-defined time horizons from radiocarbon ages is complicated by instantaneous variations in delta surface topography. However, averaged over longer periods typical of channel avulsion and migration (i.e., 1000–2000 years)^62,105,125^, a stable or growing delta system must infill accommodation generated. Therefore, the potential mass of stored sediment at any given interval will be a function of the volume created by subsidence and sea-level rise. Thus, the Holocene unit has been partitioned into Time-Equivalent depositional units (TEQ) based on regional subsidence patterns derived from a large radiocarbon database (*n* > 200) in Grall et al.^108^ and combined with eustatic sea-level reconstructions from Lambeck et al.^126^. These relative sea-level rise controls were used to map the base-level and delta surface at 6, 8, 10, and 12 ka. The resulting surfaces were spliced in GIS to form TEQ units that correspond to accommodation generated during the periods 6–0, 6–8, 8–10, 10–12 ka. To calculate the delta surface over which sediments were deposited and the volume of material stored during each TEQ, we subtracted the interpolated Holocene surface from the effective subsidence (land subsidence and sea-level rise) over those years in ArcGIS (Fig. S2 and Table S1). The volume of each TEQ was multiplied by a mean bulk density based on field measurements to derive the mass of sediment stored on the delta during each TEQ. The bulk densities used range from 1.3 g/cm^3^ in the shallow units to 1.8 g/cm^3^ in the deepest units, and a lower value of 1.1 g/cm^3^ for muddy coastal and shelf units. To calculate the grain-size distribution of each sediment package, we isolated the samples from each core contained within the TEQ, calculated the average grain-size distribution, and interpolated the grain-size distribution to the total mass of the material in each TEQ.

### Offshore and West Bengal delta regions

For the offshore and West Bengal physiographic regions where we have not collected cores, we base the budget calculations on published studies. For the offshore, growth of the subaqueous delta began after ~8 ka and the stored sediment mass is determined from seismic and core data^56,127^. Our budget calculations proportionally distribute 75% of this offshore delta sediment to the 6–0 ka interval and 25% to the 8–6 ka interval. The source of offshore sediment was partitioned between Brahmaputra (~60%) and Ganges (~40%) based on Sr measurements of shelf sediment by Garzanti et al.^128^ and Lupker et al.^129^. The grain-size distribution of sediments stored in West Bengal is taken from previously published data in Stanley and Hait^130^, who measured the mud:sand ratio to be ~70:30 in a series of Holocene-scale cores (<50 m). The average Holocene thickness of sediments in their cores was 25 m with a maximum thickness of ~45 m. Although kriging analyses show the maximum predicted thickness of Holocene sediments in West Bengal to be ~70 m, >90% of the total sediment package is stored in the upper 45 m, so the data from Stanley & Hait (2000) can be used. For comparison, our data from the Ganges–Brahmaputra interfluve east of the Ganges valley and just opposite the West Bengal region is a comparable ~63% mud and ~37% sand. Since this grain-size distribution aligns well with measurements from West Bengal^130^, we apply the same distribution for sediment stored in that region.

### Sediment surplus and deficit calculations

A simple mass-balance model compares the mass aggradation needed to offset relative sea-level rise (RSLR) for the Ganges–Brahmaputra delta with reconstructed sediment delivery rates (Fig. 3). The upper delta extends from the delta apex to the slope break at the fluvial- to tide-influenced transition (Fig. S3), the lower delta from the tidal transition at the slope break to the coast, and the marine delta from the coast to base of the subaqueous delta foresets. We apply mean subsidence rates and mean eustatic sea level to each of these regions using a bulk density of 1.5 t/m^3^ typical of the upper 20 m of sediment. Additional details on calculation steps are provided in Fig. 3.

## Supplementary information

Supplementary Information

## Data Availability

The sediment data generated in this study are provided in the Supplementary Information file. Source data are provided with this paper.

## Source data

Source Data
