# Supplementary material for: Sediment delivery to sustain the Ganges-Brahmaputra delta under climate change and anthropogenic impacts
Source: Nat Commun. 2023 Apr 27;14:2429. doi: 10.1038/s41467-023-38057-9 (PMC10140268; doi:10.1038/s41467-023-38057-9)
Supplement: Supplementary file 1 — Supplementary Information [file 41467_2023_38057_MOESM1_ESM.pdf]

SUPPLEMENTARY INFORMATION

Sediment delivery to sustain the Ganges-Brahmaputra delta under climate change and anthropogenic impacts

Jessica L. Raff [a*], Steven L. Goodbred, Jr. [a*], Jennifer L. Pickering [b], Ryan S. Sincavage [c], John C. Ayers [a], Md. Saddam Hossain [b], Carol A. Wilson [d], Chris Paola [e], Michael S. Steckler [f], Dhiman R. Mondal [h], Jean-Louis Grimaud [i], Celine Jo Grall [f,j], Kimberly G. Rogers [k], Kazi Matin Ahmed [l], Syed Humayun Akhter [m], Brandee N. Carlson [n], Elizabeth L. Chamberlain [o], Meagan Dejter [a], Jonathan Gilligan [a], Richard P. Hale [p], Mahfuzur R. Khan [l], Md. Golam Muktadir [q], Md. Munsur Rahman [r], Lauren A. Williams [a]

[a] *Department of Earth and Environmental Sciences, Vanderbilt University, Nashville, TN, USA*
[b] *Center for Applied Earth Science and Engineering Research, The University of Memphis, Memphis, TN, USA*
[c] *Department of Geology, Radford University, Radford, VA, USA*
[d] *Department of Geology and Geophysics, Louisiana State University, Baton Rouge, LA, USA*
[e] *Department of Earth and Environmental Sciences, St Anthony Falls Laboratory, University of Minnesota, Minneapolis, MN, USA*
[f] *Lamont-Doherty Earth Observatory, Columbia University, Palisades, NY, USA*
[h] *Haystack Observatory, Massachusetts Institute of Technology, Westford, MA, USA*
[i] *Centre de Géosciences, PSL University/ MINES Paris, Fontainebleau, France*
[j] *CNRS - Littoral Environnement et Sociétés, La Rochelle University, La Rochelle, France*
[k] *Institute for Arctic and Alpine Research, University of Colorado, Boulder, CO, USA*
[l] *Department of Geology, University of Dhaka, Dhaka, Bangladesh*
[m] *Bangladesh Open University, Board Bazar, Gazipur, Bangladesh*
[n] *Department of Earth and Atmospheric Sciences, University of Houston, Houston, TX, USA*
[o] *Soil Geography and Landscape Group, Wageningen University, Wageningen, Netherlands*
[p] *Department of Ocean & Earth Sciences, Old Dominion University, Norfolk, VA, USA*
[q] *Department of Environmental Science, Bangladesh University of Professionals, Dhaka, Bangladesh*
[r] *Institute of Water and Flood Management, Bangladesh University of Engineering and Technology, Dhaka, Bangladesh*

Figure S1. Grain size and bulk strontium data from Transect G sediment cores across the lower Ganges-Brahmaputra delta. Location shown in Figure 1. Upper panel plots the bulk-sediment strontium (Sr) concentration (this study), and the lower panel shows bulk sand-mud sediment distribution and calibrated radiocarbon ages (Grall et al., 2018) [1]. Also shown are Pleistocene surfaces that define the three lowstand river valleys. Note that Sr data readily distinguishes the provenance of Ganges, Brahmaputra, and Meghna sourced river sediments. Strontium values reveal the stratigraphic units of the three rivers to be largely discrete with limited mixing, enabling us to calculate the total mass of delta sediments contributed by each river.

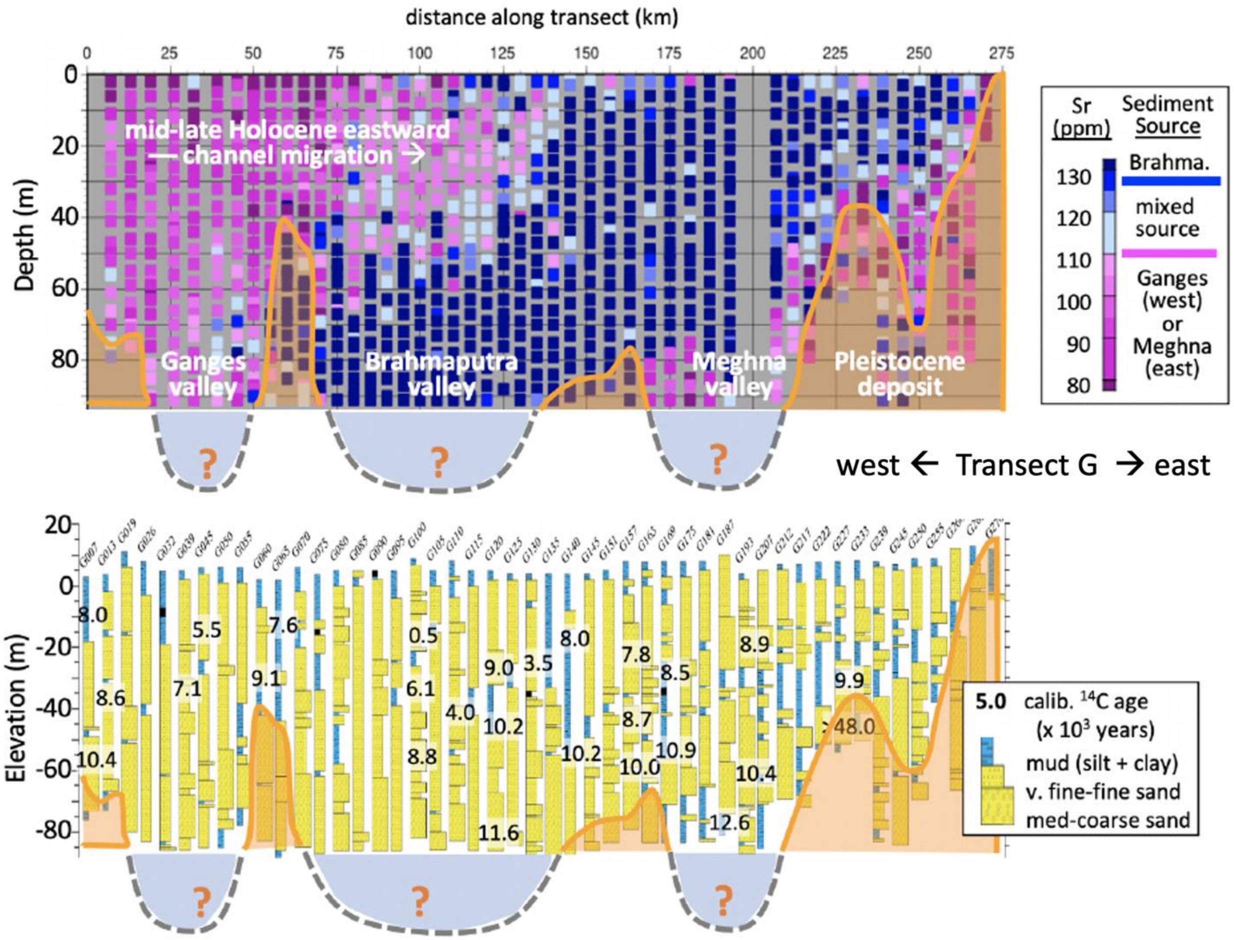

"Time-equivalent Units"

Mapping of the "time-equivalent units" (TEQ) was performed using ArcGIS. Published maps of subsidence [1] (Fig. S2) and eustatic sea level [2] were combined to reconstruct the accommodation produced by relative sea-level rise and available for infilling with delivered sediment. Extensive

prior research has shown that the sequence of deltaic facies in the G-B is dominated by aggradation (not translation), requiring that accommodation was readily infilled during Holocene sea-level rise and thus can be used to faithfully reconstruct the spatiotemporal distribution of sediment storage and serve as a proxy for fluvial sediment delivery[1,3–16]. We then calculated the areas and volumes of overlap between the Pleistocene-Holocene boundary and the reconstructed surface of relative sea-level change (i.e., TEQ) for each time unit (12–10 ka, 10–8 ka, 8–6 ka, and 6–0 ka) (Fig. S2). The Holocene-Pleistocene boundary depth was first mapped by hand using grain size, oxidation, and radiocarbon data from the BanglaPIRE dataset [1] and supplemented with additional core data from many other studies [3,9,10,17–27]. Simple co-kriging was used to create an interpolated prediction surface of the depth to the Pleistocene boundary (Fig. S3) from which the total volume of the Holocene sediment package was calculated. We then subtracted each 3D TEQ surface from the Holocene-Pleistocene boundary surface to calculate the volume of sediment stored on the delta within each time period (Table S1). We isolated the cores and samples within each TEQ to estimate the grain-size distribution of the sediment and converted sediment volume to mass using measured bulk densities (1.3-1.8 Mt/km^3) [25,28].

Figure S2. Subsidence rates and mapping of time-equivalent units (TEQ). Subsidence (A) [1] and eustatic sea level [2] were used during mapping of TEQs to estimate relative sea level changes for the Ganges-Brahmaputra delta throughout the Holocene. The delta surface during each time period is shown in blue, while red denotes the present-day extent of the delta (B-E). The World Ocean Base layer shown in panel A is from Esri, GEBCO, NOAA NGDC, HERE, Garmin, and other contributors.

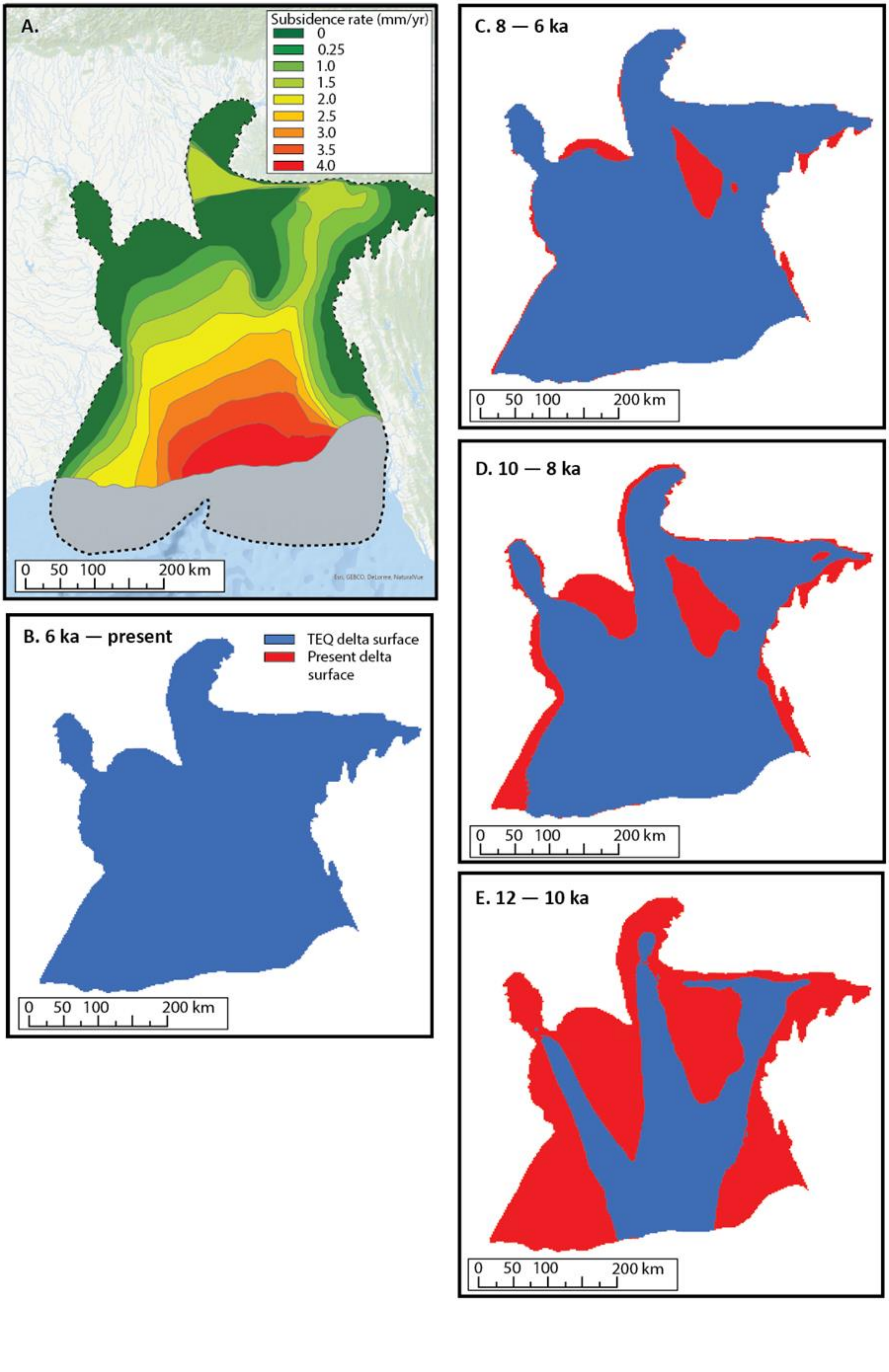

A.
Subsidence rate (mm/yr)
0
0.25
1.0
1.5
2.0
2.5
3.0
3.5
4.0
0 50 100 200 km
B. 6 ka — present
TEQ delta surface
Present delta surface
0 50 100 200 km
C. 8 — 6 ka
0 50 100 200 km
D. 10 — 8 ka
0 50 100 200 km
E. 12 — 10 ka
0 50 100 200 km

Figure S3. Interpolated surface created from core data showing depth of the Holocene-Pleistocene boundary on the onshore delta. Holocene sediment sequences are thickest in the river valley and basin settings (like Sylhet basin, NE corner of the delta), but the Pleistocene surface is only shallowly buried elsewhere on the delta, particularly near the delta apex. The red line shows the boundary between the fluvial delta (slope 10^{-4}) and the fluvial-tidal delta (10^{-5}) [12]. The World Ocean Base layer is from Esri, GEBCO, NOAA NGDC, HERE, Garmin, and other contributors.

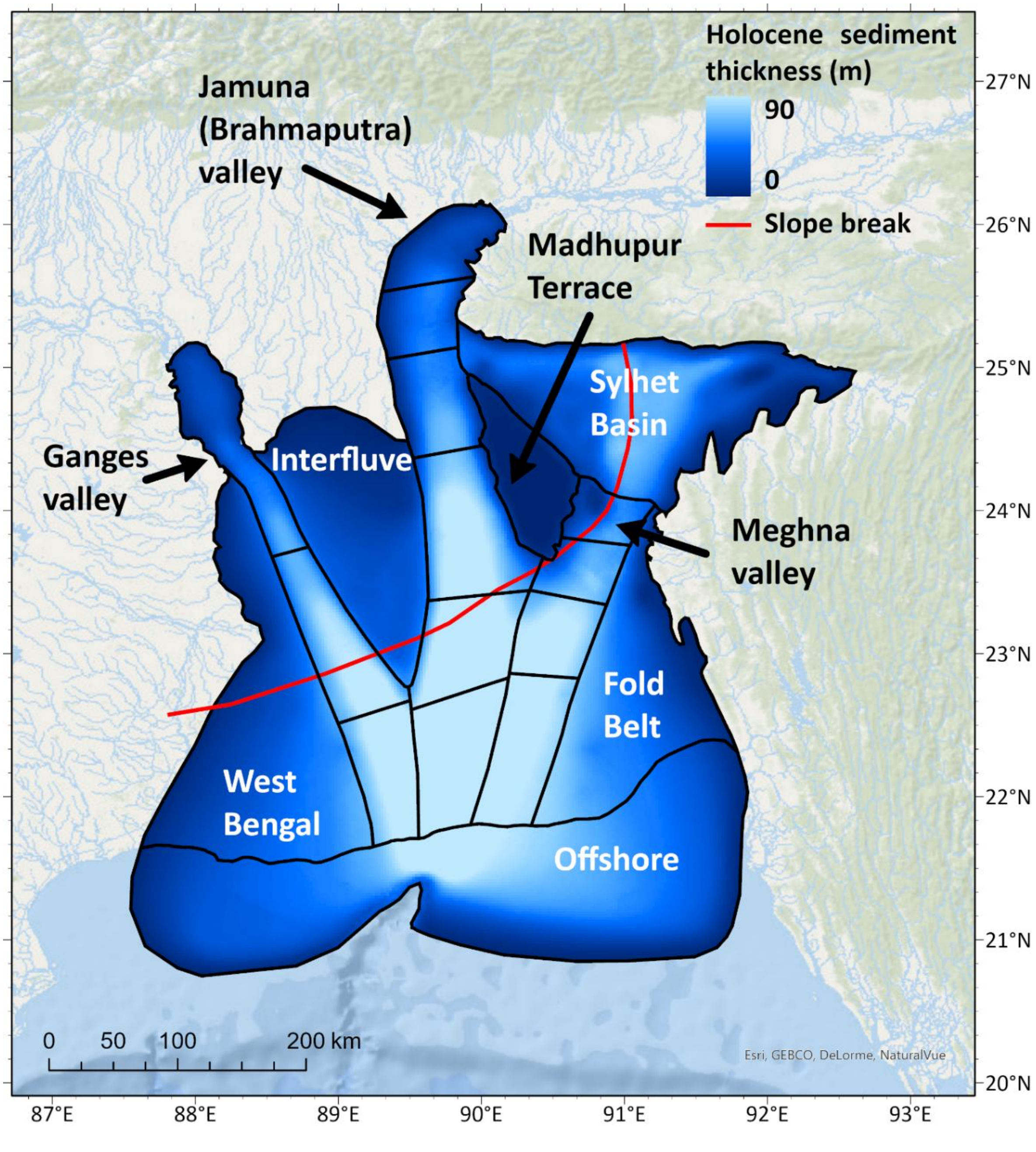

Table S1. Volume of sediment stored on the delta within each time period (TEQ). Bulk densities are relatively higher from 10–8 ka and 12–10 ka due to deep burial and a primarily sandy composition. Bulk densities for sediments deposited between 8–6 ka and 6–0 ka are relatively lower due to their shallower burial depths and to greater fraction of mass stored as less-consolidated muds in the offshore subaqueous delta.

Years	Volume (km^3)	Mean Bulk Density (Mt/km^3)	Mass (Mt)	Mass Delivery (Mt/yr)
**6-0 ka** *	4096	1238	5071156	845
**8-6 ka** *	1783	1405	2505352	1253
**10-8 ka**	2354	1700	4001800	2001
**12-10 ka**	909	1800	1636200	818

* Offshore volume distributed with 75% of offshore material contained in the 6-0 ka unit and 25% of offshore material contained in the 8-6 ka unit. Bulk density applied to offshore material is 1,100 Mt/km^3.

Reconstructed potential sediment loads

Reconstructed suspended and bedload estimates of the Holocene sediment load using the observed ratio of mud-sized sediments to sand-sized sediments in shallow (<~30 m) sediment (last 6 ka — see methods) and from Akter et al. (2016) [11] to account for preservation biases are shown in Tables S2 and S3. Thus, total fluvial sediment load during the early Holocene may have been 30–70% higher than the minimum storage rates reconstructed here, thus potentially increasing sediment delivery estimates by ~600 Mt/yr for 12–10 ka and 10–8 ka. When sediment loads are reconstructed using the suspended:bedload data from Akter et al. (2016) [11], the overall Holocene load would be ~5% higher but the last 6 ka would decrease ~25%, suggesting a preservation bias for suspended load in these younger sediments, which have not yet been buried below the depth of channel reworking. The bedload fraction drops to 45% of the stored load between 12–10 ka and 30% between 10–8 ka to ~25% between 8 ka to present. These results suggest that the Ganges-Brahmaputra delta (G-B) fluvial sediment load may be coarser than previously recognized with an average of 20-25% bedload over the Holocene. Moreover, these estimates of a coarser and larger sediment load align with sediment stored offshore (on the order of several Mt/yr), suggesting that sediment discharge rates to the G-B were likely higher than those recorded by storage alone.

Table S2. Potential sediment loads reconstructed using the ratio of total sediment (excluding bedload) and bed material estimated from Akter et al. (2016) [11]. Applying the average ratio of suspended sediment to bedload to measured bedload stored in the Holocene delta increases

the potential sediment delivery over the last 12 kyr, particularly in the early Holocene (12—8 ka).

Akter et al. (2016)			
River	Total sediment (Mt/yr)	Bed material (Mt/yr)	Total:bedload ratio
Jamuna	600	200	3
Ganges	550	200	2.75
			Average: 2.875

Observed (this study) and potential reconstructed sediment loads using Akter et al. (2016)					
Years	Observed suspended mass (Mt; <250 μm)	Observed bedload mass (Mt; 250-1000 μm)	Reconstructed suspended mass (Mt)	Potential total mass (Mt)	Potential delivery rates (Mt/yr)
6—0ka	4.10E+06	0.974E+06	2.80E+06	3.77E+06	629
8—6 ka	1.89E+06	0.618E+06	1.78E+06	2.39E+06	1197
10—8 ka	2.66E+06	1.34E+06	3.86E+06	5.20E+06	2600
12—10 ka	0.895E+06	0.741E+06	2.13E+06	2.87E+06	1436
12—0 ka	8.31E+06	3.59E+06	10.3E+06	13.9E+06	1158

Table S3. Potential sediment loads reconstructed using the ratio of mud-sized sediments to sands observed in sediments stored on the delta between 6—0 ka. Using this method, sediment loads could potentially exceed stored amounts by 20-50+%.

Observed (this study) and potential reconstructed sediment loads using the suspended:bedload ratio from 6—0 ka					
Years	Observed suspended mass (Mt; <250 μm)	Bedload mass (Mt) (Mt; 250-1000 μm)	Reconstructed suspended mass (Mt)	Potential total mass (Mt)	Potential delivery rates (Mt/yr)
6—0 ka	4.10E+06	0.974E+06	4.10E+06	5.07E+06	845
8—6 ka	1.89E+06	0.618E+06	2.45E+06	3.07E+06	1535
10—8 ka	2.66E+06	1.34E+06	4.20E+06	5.54E+06	2772
12—10 ka	0.895E+06	0.741E+06	1.66E+06	2.40E+06	1199
12—0 ka	8.31E+06	3.59E+06	12.4E+06	16.0E+06	1333

Table S4. Mass required for delta maintenance for each aggradation scenario shown in Table 3 ([a] Darby et al. (2015), *Env. Science,* **17:**1587; [b] Higgins et al. (2018), *Elementa,* **6**:20; [c] Dunn et al. (2019), *Env. Res. Letters,* **14**:084034)[29–31]

	Mass(required) (Mt/yr)		
Scenario	**low**	**med**	**high**
Modern: typical Qs	733	868	1013
Modern: low Qs	733	868	1013
paleo: 0-6 ka	426	501	563
paleo: 6-8 ka	869	1033	1224
paleo: 8-10 ka	1209	1209	1209
2050 climate only [a]	1210	1438	1710
2100 climate only [a]	1656	1971	2363
River-linking (partial) [b]	1656	1971	2363
River-linking (full) [b]	1656	1971	2363
2100 full impacts [c]	1656	1971	2363

Error analysis and uncertainties in estimates

There is no tractable process for a formal error analysis on the study's results given the many steps from collecting core samples to converting the raw data to a finished mass distribution. The propagation of errors would hold little value for assessing the accuracy of results. However, to control for uncertainty through the budget analysis, we used results from the physiographic regions with the highest data density as controls for areas with less data, such as West Bengal. These comparisons generally confirmed that the initial results were appropriate, and the few adjustments made were relatively minor and are noted in the main text. We compared our observations with those from the many studies cited in the paper, and we incorporated their data into the budget calculations wherever possible. We also evaluated uncertainty by varying key values such as the bulk density conversions and distribution of the Holocene boundary. These semi-quantitative assessments typically resulted in budget and mass deviations of 5-15%. Other known errors include small uncertainties due to clipping and other data processing in ArcGIS, although these are minor since they occur at the boundaries of the basin where Holocene sediment storage is limited by the shallow Pleistocene surface.

Although the budget may lack some precision, the overall accuracy of results appears robust. For example, we have high confidence in the major boundaries of the Holocene delta, which are well defined by highly oxidized paleosols, lowstand gravel surfaces, radiocarbon ages, and sharp upland transitions. These robust constraints mean that our total sediment calculations and mass delivery rates are well supported. In all, the sources of error and uncertainty in the budget prove to be relatively small compared to the spatial and temporal scales at which the system is being analyzed here. Based on our semi-quantitative assessments of uncertainty, we suggest that the error for any given value presented in the paper is ±10% at the 1-sigma level and ±20% at the 2-sigma level.

The greatest uncertainty may lie with the mass of sediment that bypassed the delta and was deposited either on the deep-sea Bengal Fan or advected laterally along the shelf. We evaluate this potential error in the reconstructed sediment loads presented in Tables S2 and S3, and we discuss these issues thoroughly in the main text. The potential sediment bypassing is most likely to affect values for the 12–10 ka and 10–8 ka TEQ periods, when sea level was lower and the rivers were constrained to their lowstand valleys. After 8 ka, though, there is little evidence that significant bypassing occurred once the rise of sea level slowed. Bypassing would also primarily affect the total mud mass, since fine-grained sediments will have greater transport distance and are more likely to be reworked within the delta (i.e., preservation bias). The high mud:sand ratio for the 0–6 ka and 6–8 ka, however, suggests that most of the fine-grained sediments are preserved in the delta for these periods. We also restate the key point

that any bypassing or preservation errors would only serve to increase the mass delivery rates presented here and thus simply reinforce the main points of the paper.

References

1. Grall, C. *et al.* A base-level stratigraphic approach to determining Holocene subsidence of the Ganges–Meghna–Brahmaputra Delta plain. *Earth Planet. Sci. Lett.* **499**, 23–36 (2018).

2. Lambeck, K., Rouby, H., Purcell, A., Sun, Y. & Sambridge, M. Sea level and global ice volumes from the Last Glacial Maximum to the Holocene. *Proc. Natl. Acad. Sci. U. S. A.* **111**, 15296–15303 (2014).

3. Shamsudduha, M., Uddin, A., Saunders, J. A. & Lee, M. K. Quaternary stratigraphy, sediment characteristics and geochemistry of arsenic-contaminated alluvial aquifers in the Ganges-Brahmaputra floodplain in central Bangladesh. *J. Contam. Hydrol.* **99**, 112–136 (2008).

4. Goodbred Jr., S. L. *et al.* Piecing together the Ganges-Brahmaputra-Meghna river delta: Use of sediment provenance to reconstruct the history and interaction of multiple fluvial systems during Holocene delta evolution. *Bull. Geol. Soc. Am.* **126**, 1495–1510 (2014).

5. Steckler, M. S. *et al.* Synthesis of the distribution of subsidence of the lower Ganges-Brahmaputra Delta, Bangladesh. *Earth-Science Rev.* **224**, (2022).

6. Paszkowski, A., Goodbred, S., Borgomeo, E., Khan, M. S. A. & Hall, J. W. Geomorphic change in the Ganges–Brahmaputra–Meghna delta. *Nat. Rev. Earth Environ. 2021* 1–18 (2021). doi:10.1038/s43017-021-00213-4

7. Krien, Y. *et al.* Present-day subsidence in the Ganges-Brahmaputra-Meghna Delta: eastern amplification of the Holocene sediment loading contribution. *Geophys. Res. Lett.* **46**, 1–9 (2019).

8. Karpytchev, M. *et al.* Contributions of a Strengthened Early Holocene Monsoon and Sediment Loading to Present-Day Subsidence of the Ganges-Brahmaputra Delta. *Geophys. Res. Lett.* **45**, 1433–1442 (2018).

9. Khan, S. R. & Islam, B. Holocene stratigraphy of the lower Ganges-Brahmaputra river delta in Bangladesh. *Front. Earth Sci. China* **2**, 393–399 (2008).

10. Sarkar, A. *et al.* Evolution of Ganges-Brahmaputra western delta plain: Clues from sedimentology and carbon isotopes. *Quat. Sci. Rev.* **28**, 2564–2581 (2009).

11. Akter, J., Sarker, M. H., Popescu, I. & Roelvink, D. Evolution of the Bengal Delta and Its Prevailing Processes. *J. Coast. Res.* **321**, 1212–1226 (2016).

12. Wilson, C. A. & Goodbred Jr., S. L. Construction and Maintenance of the Ganges-

Brahmaputra-Meghna Delta: Linking Process, Morphology, and Stratigraphy. *Ann. Rev. Mar. Sci.* **7**, 67–88 (2015).

13. Goodbred Jr., S. L. & Kuehl, S. A. Holocene and modern sediment budgets for the Ganges-Brahmaputra river system: Evidence for highstand dispersal to flood-plain, shelf, and deep-sea depocenters. *Geology* **27**, 559–562 (1999).

14. Goodbred Jr., S. L. & Kuehl, S. A. Enormous Ganges-Brahmaputra sediment discharge during strengthened early Holocene monsoon. *Geology* **28**, 1083–1086 (2000).

15. Goodbred Jr., S. L. & Kuehl, S. A. The significance of large sediment supply, active tectonism, and eustasy on margin sequence development: Late Quaternary stratigraphy and evolution of the Ganges–Brahmaputra delta. *Sediment. Geol.* **133**, 227–248 (2000).

16. Goodbred, S. L., Kuehl, S. A., Steckler, M. S. & Sarker, M. H. Controls on facies distribution and stratigraphic preservation in the Ganges-Brahmaputra delta sequence. *Sediment. Geol.* **155**, 301–316 (2003).

17. Hoque, M. A., McArthur, J. M. & Sikdar, P. K. The palaeosol model of arsenic pollution of groundwater tested along a 32km traverse across West Bengal, India. *Sci. Total Environ.* **431**, 157–165 (2012).

18. Michels, K. H., Kudrass, H. R., Hübscher, C., Suckow, A. & Wiedicke, M. The submarine delta of the Ganges-Brahmaputra: Cyclone-dominated sedimentation patterns. *Mar. Geol.* **149**, 133–154 (1998).

19. Palamenghi, L., Schwenk, T., Spiess, V. & Kudrass, H. R. Seismostratigraphic analysis with centennial to decadal time resolution of the sediment sink in the Ganges-Brahmaputra subaqueous delta. *Cont. Shelf Res.* **31**, 712–730 (2011).

20. Pickering, J. L. *et al.* Impact of glacial-lake paleofloods on valley development since glacial termination II : A conundrum of hydrology and scale for the lowstand Brahmaputra-Jamuna paleovalley system. *GSA Bull.* **131**, 58–70 (2018).

21. Ahmed, M. K., Islam, S., Rahman, M. S., Haque, M. R. & Islam, M. M. Heavy Metals in Water , Sediment and Some Fishes of Buriganga River, Bangladesh. *Int. J. Environ. Res.* **4**, 321–332 (2010).

22. BADC (Bangladesh Agricultural Development Corporation). Deep Tubewell II Project: Final Reports. (1992).

23. Pate, R. D., Goodbred, S. L. & Khan, S. R. Delta Double-Stack: Juxtaposed Holocene and Pleistocene Sequences from the Bengal Basin, Bangladesh. *Sediment. Rec.* **7**, 4–9 (2009).

24. DPHE-JICA (Department of Public Health Engineering and Japanese International Cooperation Agency). *Development of deep aquifer database and preliminary deep*

aquifer map. Final Report. (2006).

25. (JICA), J. I. C. A. *Vol. 6: Geology and Stone Material, Part 1: Geology*. *People's Republic of Bangladesh Jamuna River Bridge Construction Project: Feasibility study report* (1976).

26. Ghosal, U., Sikdar, P. K. & McArthur, J. M. Palaeosol Control of Arsenic Pollution: The Bengal Basin in West Bengal, India. *Groundwater* **53**, 588–599 (2015).

27. Hait, A. K. *et al.* New dates of Pleisto-Holocene subcrop samples from South Bengal, India. *Indian J. Earth Sci.* **23**, 79–82 (1996).

28. Kuehl, S. A., Levy, B. M., Moore, W. S. & Allison, M. A. Subaqueous delta of the Ganges-Brahmaputra river system. *Mar. Geol.* **144**, 81–96 (1997).

29. Darby, S. E. *et al.* A first look at the influence of anthropogenic climate change on the future delivery of fluvial sediment to the Ganges–Brahmaputra–Meghna delta. *Environ. Sci. Process. Impacts* **17**, 1587–1600 (2015).

30. Higgins, S. A., Overeem, I., Rogers, K. G. & Kalina, E. A. River linking in India: Downstream impacts on water discharge and suspended sediment transport to deltas. *Elementa* **6**, (2018).

31. Dunn, F. E. *et al.* Projections of declining fluvial sediment delivery to major deltas worldwide in response to climate change and anthropogenic stress. *Environ. Res. Lett.* **14**, 084034 (2019).
